# State-of-the-Art Research on Diabetic Retinopathy

**DOI:** 10.3390/jcm11133790

**Published:** 2022-06-30

**Authors:** Rafael Simó

**Affiliations:** 1 Diabetes and Metabolism Research Unit, Vall d’Hebron Research Institute (VHIR), Autonomous University of Barcelona, Vall d’Hebron Campus, 08035 Barcelona, Spain; rafael.simo@vhir.org; 2 Centro de Investigación Biomédica en Red de Diabetes y Enfermedades Metabólicas Asociadas (CIBERDEM), Instituto de Salud Carlos III (ICSIII), 28029 Madrid, Spain

The scientific community and health care providers should be seriously worried by the fact that diabetic retinopathy (DR) remains the main cause of preventable blindness in the working age population. This is a common sentence in the introduction section of papers on this issue and reflects the low efficiency of translational research in this field. For this reason, I was happy to lead this Special Issue, “State-of-the-Art Research on Diabetic Retinopathy”, and very grateful for the excellent contributions in either original articles or critical reviews of several renowned researchers of DR.

There is no doubt that screening for DR is a crucial factor for preventing a sight-threatening eye disease. However, since the Sant Vincent Declaration in 1989, the fight against diabetic complications should move rapidly, but this has not been the case. In fact, the procedures involved in the identification of DR have not significantly changed worldwide and particularly in low-income countries. Nevertheless, the recent irruption of telemedicine, artificial intelligence, and portable fundus cameras are facilitating the screening of DR and improving its capability with respect to cost–benefit. In this regard, the paper by Agrawal et al. [1] showing the willingness of patients with diabetes to use telemedical technology, particularly if it is recommended by the physician and provided without additional costs, provides a great deal of value to implementing these technologies in the real world. Another important point is the prioritization of screening for DR. This is not a minor point in a highly prevalent disease such as diabetes. There is evidence that depending on the risk factors the screening can be performed every two years instead of every year as has been classically recommended. In this Special Issue, Wang et al. [2] develop an accurate and cost-effective predictive model and an easy-to-use risk index to identify patients with a high risk for DR and to counsel them for an ophthalmic examination, thus improving the compliance for the early detection of DR.

The advancement of retinal imaging has been tremendous in recent years. One of the most useful advances has been ultrawide field imaging (UWF). This method enables the visualization of a significantly greater area of the retina, thereby permitting us to identify the lesions that are located mainly in the peripheral retina, which were overlooked with previous standard approaches. Additionally, in this issue, Ashraf et al. [3] provide a comprehensive and critical review based on their own experience of UWF and examine the role of UWF alone or combined with fluorescein angiograms towards improving DR classification. Optical coherence tomography angiography (OCTA) has been another main driver of the imaging revolution for the study of DR. Apart from a more exhaustive and accurate examination of retinal microcirculation, the information obtained by OCTA, such as vessel density or foveal avascular zone (FAZ), could be useful for identifying patients more prone to developing systemic complications related to ischemia (i.e., coronary disease, stroke, etc.). In this Special Issue, Alé-Chilet et al. [4] deal with the potential role of OCTA in identifying patients with type 1 diabetes at a higher risk for diabetic kidney disease or its progression. This study is an example of a new research area based on the use of retinal imaging beyond the eye to define new phenotypes and to improve the risk stratification for the systemic complication of diabetes. To complete the advances in retinal imaging, Vujosevic et al. [5] provide evidence that quantitative color fundus autofluorescence could be useful for the metabolic evaluation of the diabetic retina. This study opens a new avenue for using retinal imaging as a tool for monitoring the short-term effects of metabolic control in a personalized manner. 

The treatment of DR is another topic that needs urgent breakthroughs. The recent assumption that neurodegeneration plays a role in the pathogenesis of a significant proportion of patients with diabetes has led to the development of new therapeutic strategies based on neuroprotection, which could be initiated in the early stages of the disease [6,7]. Nevertheless, current approved treatments, such as laser photocoagulation or intravitreal injections of antiangiogenic factors or corticosteroids, are only addressed to the advanced stages of DR. In this Special Issue, there are three articles that show interesting results in the setting of DR treatment. Lois et al. [8] demonstrate that the subcutaneous administration of cibinetide, a synthetic peptide derived from the structure of the B helix of EPO with important antiapoptotic, anti-inflammatory, and anti-permeability effects, was safe after a 12-week treatment. Although larger studies testing its effectiveness are required, this is an interesting result because EPO is a powerful neuroprotective factor synthesized by the retina, and because EPO receptor has also been reported to be abundant in retinal pigment epithelium and neuroretina [9]. In another enlightening article, Enright et al. [10] provide evidence that the beneficial effects of fenofibrate in a spontaneous diabetes model (db/db mouse) are not related to its capacity to induce the expression of PPARα-target genes in the whole retina or Müller glia. Since fenofibrate could be a potential drug for treating DR, this intriguing finding deserves further research. Frizziereo et al. [11] review subthreshold micropulse laser treatment as a therapeutic approach to diabetic macular edema and conclude that this laser modality seems to determine a long-term normalization of specific retinal neuroinflammation. Therefore, it is possible that those patients with more glial activation/inflammation, which might be identified by means of a liquid biopsy of aqueous humor, could be the most appropriate candidates for this laser modality.

As is generally the case in science, basic research is the backbone for nourishing innovation and usually provides us with a myriad of proof of concepts to be tested in the clinical setting. In this Special Issue, Fresia et al. [12] presented an elegant experimental work showing the deleterious effects of hypoglycemia on the neuroretina and the underlying mechanisms. This is very interesting because the published information regarding the effects of hypoglycemia on the neuroretina is scarce and cannot be extrapolated to the abundant reports on this issue that exist on the brain. In addition, this finding supports the concept of promoting the use of drugs with a low capacity for provoking hypoglycemia, but specific clinical trials aimed at examining this subject are still needed. High-throughput technologies such as proteomics of the vitreous fluid are accelerating the knowledge of the complex and intricated pathogenesis of DR. Weber et al. [13] share a suggested protocol for such studies and propose next steps for moving the field forward. In addition, the authors recommend that published vitreous proteomic studies should always be deposited in a freely accessible public repository, such as PRIDE (https://www.ebi.ac.uk/pride/ (accessed on 22 May 2021)) or Peptide Atlas (http://www.peptideatlas.org (accessed on 22 May 2021)). Finally, the discovery of new targets for treating DR that reach regulatory clinical approval is a challenge that needs to be met. In this Special Issue, Nath. et al. [14], using primary Müller glial cells, demonstrated that HspB4/αA-crystallin modulates multiple key inflammatory pathways, which thereby suggests its potential as a therapeutic target for diabetes induced neuroinflammation. In addition, Kim et al. [15] provide the first piece of evidence that hyperglycemia upregulates Rab20 expression in retinal endothelial cells and Müller cells. Moreover, this aberrant Rab20 upregulation compromises gap junction intercellular communication (GJIC) activity and promotes the loss of the retinal endothelial cells and retinal Müller cells associated with DR. Therefore, reducing Rab20 expression could be a useful strategy for preventing hyperglycemia-induced vascular and Müller cell death. Last but not least, Pan et al. [16] show how the application of systems biology using the investigative approaches currently applied to diabetic kidney disease can promote a more thorough understanding of the structure, function, and progression of DR. This holistic manner of acquiring information might enable the discovery of new targeted therapies.

In summary, this Special Issue provides us with a brief overview of some of the aspects of the main topics in the research on DR, which is authored by reputed researchers. The quick and abundant responses from the authors warrants a second edition of “State-of-the-Art Research on Diabetic Retinopathy”. At present, I can only thank you in advance for all your eventual collaborations and propose a toast, because “the era of personalized medicine in the field of DR has begun”.
